# Combining advanced 3D spheroid-based skin models with deep-learning-based image analysis enables in-depth investigation of keratinocyte differentiation and barrier function

**DOI:** 10.3389/fbioe.2026.1887652

**Published:** 2026-09-09

**Authors:** Tiziana Cesetti, Claudia Buerger, Nathalie Couturier, Elina Nuernberg, Roman Bruch, Mario Vitacolonna, Victoria Lang, Mathias Hafner, Markus Reischl, Torsten Fauth, Rüdiger Rudolf

**Affiliations:** 1 Institute of Molecular and Cell Biology, Technische Hochschule Mannheim, Mannheim, Germany; 2 CeMOS Research and Transfer Center, Technische Hochschule Mannheim, Mannheim, Germany; 3 Department of Dermatology, Venereology and Allergology, Goethe University Frankfurt, University Hospital, Frankfurt, Germany; 4 Institute for Automation and Applied Informatics, Karlsruhe Institute of Technology, Karlsruhe, Germany; 5 BRAIN Biotech AG, Zwingenberg, Germany; 6 Akribion Therapeutics GmbH, Zwingenberg, Germany

**Keywords:** deep-learning, differentiation, functional barrier, keratinocytes, nuclei, segmentation, single-cell analysis, spheroids

## Abstract

To date, organotypic skin models represent the gold standard for preclinical dermatological and toxicological studies. However, they are variable in quality and require long maturation times and many cells, mainly of primary origin. We propose dermal-epidermal spheroids as an alternative model that balances the physiological relevance and throughput. Alongside the corresponding full thickness skin models, three different fibroblast/keratinocyte coculture spheroids were generated. These studies used the commonly employed HaCaT cells as well as two recently immortalized keratinocyte cell lines, NHK-SV/TERT and NHK-E6/E7. To investigate their differentiation with detailed spatiotemporal resolution, a deep-learning segmentation-based pipeline capable of revealing nuclear morphology and positioning, as well as marker expression with single-cell precision, was developed and applied. Moreover, the formation of a functional barrier was assessed by live imaging of Lucifer Yellow diffusion. NHK-based coculture spheroids displayed strong evidence of functional maturation, including stratification and aspects of cornification and barrier formation, closely recapitulating the features of the corresponding full-thickness models. Furthermore, NHK-E6/E7 cells showed to be the most and HaCaT cells the least suitable alternative to primary keratinocytes in both spheroids and full thickness models. Given their scalability and compatibility with automation, micro-skin fibroblast/NHK-based 3D coculture spheroids might represent a promising new platform for pharmaceutical, cosmetic, and toxicological testing.

## 1 Introduction

The skin, the largest organ in the human body, serves as a dynamic interface with the environment. Its complex structure fulfills essential functions, including sensory perception, thermoregulation, and barrier formation, to protect the body from UV irradiation, dehydration, and chemical and microbial insults. To maintain these functions, the outermost layer of the epidermis is continuously renewed by the proliferation of keratinocytes in the basal layer and their orderly differentiation and maturation into corneocytes in the apical layers. Each epidermal layer is characterized by the expression of specific markers. Cytokeratin 14 (KRT-14) is primarily expressed in basal keratinocytes, marking the proliferative layer. Cytokeratin 10 (KRT10), a marker of early keratinocyte differentiation, is abundant in the lower spinous layer up to the granular layer, whereas involucrin (IVL) is expressed in the upper stratum spinosum and increases in the stratum granulosum (Watt, 1983). Filaggrin is confined to the stratum granulosum, where it contributes to the formation of the cornified envelope and skin barrier (Watt, 1983).

Beneath the epidermis lies the dermis, consisting of fibroblasts embedded in collagen and elastin fibers that provide strength and elasticity to the skin. The simplest human epidermal models, monolayer cultures of keratinocytes or fibroblasts, are attractive because of their short cultivation time and low cost. However, they do not recapitulate the complexity of the multilayered epidermis and many of its physiological functions. Animal testing, however, presents challenges in translating to human skin and raises ethical concerns. Thus, various human 3D skin equivalent models have been developed over the past few decades and used for preclinical studies in dermatology, toxicology, and penetration testing (Klicks et al., 2017; Ribeiro et al., 2025). Simplified epidermal models can be generated by cultivating keratinocytes on an artificial substrate and inducing differentiation through exposure to the air-liquid interface (ALI) (Pruniéras et al., 1983). In contrast, full-thickness skin models (FTSMs) are more advanced constructs, including a dermal layer consisting of matrix-embedded fibroblasts overlaid by keratinocytes that mature into a stratified epidermis. When using primary keratinocytes isolated from human skin, reconstituted models closely resemble native skin, displaying a stratified and cornified epithelium and functional barrier (Boyce et al., 1990; Stark et al., 2004; Niehues et al., 2018). However, primary keratinocytes show high donor-dependent variability, a short lifespan, and limited availability (van Drongelen et al., 2014; Reijnders et al., 2015). A common alternative is the spontaneously immortalized cell line HaCaT (Boukamp et al., 1988), which exhibits limited differentiation and is unable to form a functional epidermal barrier (Boelsma et al., 1999; Moran et al., 2021). To overcome these issues, several immortalized keratinocyte cell lines have been generated in the past (Dickson et al., 2000; Ramirez et al., 2003; Chapman et al., 2010; Choi et al., 2017; Kim et al., 2017; Beilin et al., 2021) which perform better at mimicking the characteristics of primary keratinocytes, as well as in the generation of 3D epidermal or full-thickness skin equivalents (Dickson et al., 2000; Chapman et al., 2010; van Drongelen et al., 2014; Smits et al., 2017). We have recently shown that the commercially available cell line NHK-SV/TERT (NHEK/SV-TERT3-5, Evercyte GmbH) (Wagner et al., 2018; Weinmuellner et al., 2018) and our own cell line NHK-E6/E7 (Ritzmann et al., 2023) can reconstitute a stratified epidermis and functional epidermal barrier in FTSMs (Jahn et al., 2024).

Despite this progress, *in vitro* skin models face several challenges, including their labor- and time-intensive manufacturing, the need for a large number of cells, and the lack of robust automated procedures for their generation and readout analysis (Czyz et al., 2023). These limitations have restricted the use of *in vitro* skin models in high-throughput approaches, impeding their implementation in early drug screening. Self-assembling 3D cultures, termed spheroids, have been introduced in cancer research as a compromise between physiological relevance and throughput (Sutherland et al., 1971). Spheroids can be easily and rapidly obtained in large quantities with homogeneous compositions, sizes, and growth behavior (Vitacolonna et al., 2024a). Typically, they are composed of a few thousand cells with a total diameter of a few hundred micrometers. They can consist of a single cell type or different cell types and are superior in terms of physiological relevance compared to 2D adherent cell cultures (Pampaloni et al., 2007; Duval et al., 2017). Typically, spheroid formation is achieved by spontaneous cell aggregation in cell-repellent U-shaped 96-well plates (Klicks et al., 2019; Schäfer et al., 2021), agarose-coated plates (Woappi et al., 2020; Bila et al., 2024) or hanging drops (Vollmers et al., 2012). Although their academic and industrial applications are rapidly expanding, especially in cancer research (Lin and Chang, 2008; Costa et al., 2016; Liu et al., 2021; Jiang et al., 2023; Rodrigues et al., 2024; Strzyga-Łach et al., 2024), the use of spheroids in the field of dermatology remains relatively limited (Choi and Lee, 2015; Klicks et al., 2017; Klicks et al., 2019; Schäfer et al., 2021; Ritzmann et al., 2023; Chen et al., 2025). Thus, we aimed to establish a new full-skin 3D culture system that is suitable for higher throughput by generating coculture spheroids of fibroblasts with different keratinocyte lines. Their regular and reliable growth characteristics have facilitated quantitative analysis. Therefore, we quantitatively compared their stratification, morphology, and barrier function to classical *in vitro* skin models. To this end, we developed a novel analytical tool set based on advanced image analysis and deep learning. Our data indicate that spheroids made of fibroblasts and either NHK-SV/TERT or NHK-E6/E7 cells easily formed and exhibited a clear stratification, formation of tight junctions, and a functional barrier, with NHK-E6/E7 spheroids performing the best. Together, these factors might arguably render such spheroids as a novel dermatological test model with intermediate complexity, physiological relevance, and throughput.

## 2 Materials and methods

### 2.1 Cell cultivation

*CCD-SK1137 Fibroblasts*: CCD-1137Sk human fibroblasts were from American Type Culture Collection (ATCC, Manassas, VA, United States) and were cultivated in IMDM supplemented with 10% FBS at 13 × 10^4^ cells/cm^2^ density. *HaCaT keratinocytes:* HaCaT cells were obtained from CLS Cell Line Service GmbH (Eppelheim, Germany) and cultivated in DMEM medium with Na-pyruvate (1 mM) and glutamine (4 mM) (SIGMA D5796) supplemented with 10% FBS. The cells were grown in plastic flasks (Greiner) at a density of 5 × 10^3^ cells/cm^2^. *NHK/SV-TERT3-5:* were purchased from EverCyte GmbH (Austria). Cells were obtained from human epidermal keratinocytes by transfection with a plasmid encoding the SV40 early region, followed by transduction with a retroviral expression vector (pLXSN) containing the TERT gene. *NHK-E6/E7:* were provided by BRAIN Biotech AG (Germany). Juvenile keratinocytes were immortalized via lentiviral transfection with HPV16-NHK-E6/E7. The latter two cell lines were cultivated in 2D-KGM medium (C-20011, Promocell), containing bovine pituitary extract (30 μg/mL), EGF (human recombinant, 0.125 ng/mL), insulin (human recombinant, 5 μg/mL), hydrocortisone (0.33 μg/mL), epinephrine (0.39 μg/mL), transferrin (human recombinant, 10 μg/mL), and CaCl_2_ (0.06 mM), at a density of 6,000–8,000 cells/cm^2^.

### 2.2 Generation of 3D-Full thickness skin model (FTSM)

FTSM were generated according to Idrees et al. (2021). Briefly, 5 × 10^4^ CCD-SK1137 fibroblasts were grown in a collagen matrix for 7 days in 12-well 0.4 μm pore size ThinCerts with PET-membranes (Greiner Bio-One GmbH, Frickenhausen, Germany), which were overlaid with IMDM Medium + 10% FBS. Keratinocytes (5 × 10^5^) were seeded in CnT-Prime medium (CELLnTEC; Bern, Switzerland). After 3 days, the medium was changed to differentiation medium (3 parts CnT-Prime 3D Barrier Medium (CELLnTEC)/ 2 parts DMEM)). The next day, the inserts were transferred to deep-well plates and lifted to the air-liquid interface (ALI) to induce keratinocyte maturation, as indicated by keratinization and stratification. The medium was changed every other day, and the models were harvested after 10 days at ALI. For barrier function studies, 1 mM Lucifer yellow (Sigma-Aldrich) was applied to top of the model for 60 min at 37 °C.

### 2.3 Generation of spheroid cocultures

CCD-SK1137 fibroblasts were seeded with 150 µL of IMDM + 10% FBS medium in BIOFLOAT™ 96-well plates (faCellitate, Mannheim, Germany) at a density of 5 × 10^3^ cells/well centrifuged at 600 rpm for 5 min (D −3). After 3 days, 100 µL of the medium was removed, and keratinocytes (5 × 10^3^ cells/well) were added to 100 µL 2D-KGM medium (CELLnTEC) (D 0) and centrifuged. The medium (100 µL) was changed on D1 and D3 using differentiation medium (3 parts CnT-Prime 3D Barrier Medium (CELLnTEC)/ 2 parts DMEM)). Coculture spheroids were collected after 2, 4, or 6 days and processed for immunostaining.

### 2.4 Histochemistry

Sections were stained with Mayer´s Hämalaun/Hämatoxylin (5 min; Sigma-Aldrich, 51275) and with Eosin acid 0.5% (5 min; Sigma-Aldrich, 1.09844) solutions, dehydrated, and mounted with Entellan (Roth).

### 2.5 Immunostaining

*FTSM*: The membrane was cut from the insert, fixed in formalin, and embedded in paraffin. Sections (4 µm) were prepared and stained with hematoxylin and eosin (HE) or used for immunohistochemical staining. For immunostaining, sections were de-paraffinized and heat-induced epitope retrieval was executed in citrate buffer (10% HIER Citrate Buffer pH 6.0 Zytomed, Berlin, Germany) for 20 min at 95 °C. Staining with the following antibodies was performed overnight at 4 °C with primary antibody or concentration adjusted isotype control antibody in Signalstain buffer (CellSignaling): rabbit anti-IVL (1:1000; Abcam, ab20202), mouse anti-KRT14 (1:5000; BioRad, MCA890), rabbit anti-KRT10 (1:5000; Abcam, ab76318), rabbit anti-FLG (1:200; LSBio, LS-C751132), rabbit anti-CLD-1 (1:500; Thermofisher, 51-9000). Secondary antibodies were applied at 1:1000 for 1 h (anti-mouse Cy5 labelled and anti-rabbit TxRed labelled, ThermoFisher). Sections were stained with DAPI and mounted with Fluoromount G.

*Spheroids*: Spheroids were collected and fixed for 30 min at 37 °C in PFA 4%. They were then washed with PBS and stained with DRAQ5 (Thermo Fisher Scientific). After a sequential dehydration in 15% sucrose/PBS overnight, followed by 40% sucrose/PBS for 4 h at 4 °C, spheroids were embedded in OTC cryomedium (Leica Biosystems) and deep-frozen. A CM-1950 cryostat (Leica Biosystems) was used to prepare 15 μm-thick slices. The sections were fixed on microscope slides, washed with PBS, permeabilized with 0.1% Triton X-100 in PBS for 5 min, followed by blocking with BSA 3% in PBS for 1 h at RT. Staining was performed incubating overnight at 4 °C with the following primary antibodies: rabbit anti-IVL (1:1000; Abcam, ab20202), mouse anti-KRT14 (1:1000; Thermofisher, PA5-16722), rabbit anti-KRT10 (1:1000; Abcam, ab76318), mouse anti-FLG (1:100; SantaCruz, sc-66192, AKHa1), mouse anti-CLD-1 (1:100; Thermofisher, 51-9000), rabbit anti-collagen IV (1:400; Biomol, 600-401-106-0.5), rabbit anti-cleaved caspase 3 (1:400; Cell Signalling, 9661), diluted to the appropriate concentration in 3% BSA/PBS. After washing with PBS, the slices were incubated with the following secondary antibodies (all 1:1000) for 3 h at RT: donkey anti-mouse Alexa-Fluor 488 (Thermofisher, A2102), donkey anti-rabbit Alexa Fluor 555 (Thermo Fisher, A32794). The nuclei were counterstained with DRAQ5 (Thermo Fisher, 62251). The sections were mounted using Mowiol (Roth 0713.2).

### 2.6 Microscopy

*FTSM and spheroids histochemistry imaging*: Images were acquired with the upright Slide Scanner Aperio Versa 8 equipped with a Leica ×20 objective 0,55/DRY and using the software Aperio Versa 1.0.4.125.

*FTSM immunofluorescence imaging:* Images were acquired using a Nikon Eclipse Ci-L microscope and NIS Elements software with a ×20 CFI Plan Apochromat objective at 1, 750 × 1, 000 pixels.

*Spheroid confocal fluorescence images:* were acquired with an inverted Leica TCS SP8 confocal microscope (Leica Microsystem CSM, Mannheim, Germany) equipped with an HC APO 20x/0.75 IMM CORR objective, using Leica Application Suite X software and Immersion Type G (Leica Microsystem, RI 1.47). All images were acquired using the following settings: resolution 1024x1024 pixels, bidirectional acquisition, scan frequency 400 Hz, line average 2x, z-step of 2 μm; laser intensity 1.2% for 488 nm, 1% for 555 nm, and 1.7% for 633 nm, and gain 712, 780, and 652, respectively.

*Live Imaging – confocal*: Spheroids were loaded with CellMask Deep Red (1:1000; Thermofisher, C10046) in medium for 1 h at 37 °C, then washed 2 times isotonic buffer (329 mOsm; concentrations in mM: KCl 5, MgCl_2_ 1, CaCl_2_ 2, HEPES 10, NaCl 145, glucose 10). To impair the cell barrier, half of the spheroids were treated with 0.1% SDS at 37 °C for 30 min. Spheroids were then placed on a µ-Slide III 3D Perfusion slide (ibidi) and fixed with Scaffolene (gelatin nonwoven CL130 scaffold pads high density – crosslinked, 130 g/m^2^ from Freudenberg, perforated with a classical hole puncher). The slide was connected, using Luer-lock adaptors and tubing, to syringes containing either isotonic solution or 1 mM LY (SIGMA, L0144). The speed of gravity-mediated perfusion was 1.3 mL/min; complete solution exchange in the µ-well required 5–15 s. After recording for 1.5 min with isotonic buffer perfusion, LY was applied for 3.5 min as indicated, after which we switched to isotonic solution perfusion. Confocal imaging was performed using the following settings. For CellMask Deep Red: 638 nm laser, PMT 640-789, gain 650. For LY: 405 nm laser, PMT 600-600, gain 800. Three z-planes were imaged every 6 s at depths of 0, 35, and 70 µm.

### 2.7 Image analysis

*Fiji Immunostaining Analysis*: For the 3D-Full Thickness model, the keratinocyte region was defined based on the KRT14/Lucifer Yellow merged image. Here, the fibroblast/keratinocyte boundary was defined based on the KRT14 signal, whereas the keratinocytes/air boundary was defined based on the LY signal. This ROI was then applied to all channels; the signal outside this region was set to zero, and the mean intensity was measured only within the keratinocyte region. To analyze the depth penetration of LY, a vertical ROI (12 µm × 120 µm) was applied to the LY image in a representative region, and the mean intensity was measured along the y-axis. The mean intensity was plotted versus the distance from the outer border (the keratinocyte/air boundary was set at a distance of 0). The image intensity profiles were normalized and smoothed using a Savitzky–Golay filter to reduce noise while preserving the peak shape. Peaks were automatically identified based on the minimum height and prominence thresholds. The position and height of each peak were determined, and the full width at half maximum (FWHM) was calculated to estimate peak sharpness. Linear regression was applied to the ascending and descending flanks to quantify the slope characteristics. The area under the curve (AUC) was determined using trapezoidal integration across the FWHM boundaries, and a slope change point downstream of the peak was used to define the full peak width. All analyses and visualizations were implemented in Python (NumPy, SciPy, pandas, and Matplotlib), and the quantitative results were exported to Excel for further evaluation.

For spheroids, the raw confocal data were converted to multi-channel.tif files using Fiji. For each spheroid, three consecutive z-projections with the best signal were summed and used for the quantitative analysis. Using the wand-tracing tool, we manually selected the outer border of the spheroid based on the involucrin-positive signal, whereas the fibroblast core region was based on the KRT14-negative region. The fibroblast region was subtracted from the whole-spheroid region to obtain the keratinocyte region and the area (kerA) was measured. The keratinocyte region was applied to all channels. The “Normalized Integrated Density” for the KRT14, KRT10, involucrin and Claudin-1 signal in the keratinocytes regions was determined as follows. By setting the lower threshold limit to 30, we defined the positive region where we measured the area (posA) and mean intensity (MI). We normalized the positive area to the keratinocyte area (NA=(posA/kerA) × 100) and multiplied it with the mean intensity (Normalized Integrated Density = NA × MI).

For the Lucifer Yellow Live Imaging of spheroids, the CellMask Deep Red signal was used to define the whole spheroid region by thresholding, which was then applied to the LY channel. If the Scaffolene was visible within the spheroid region, the Scaffolene signal was deleted in the LY channel to avoid its contribution. At each time point, we measured the mean intensity in the spheroid region (MI_sph_) and in a smaller ROI outside the spheroids (MI_back_). MI_sph_ was normalized to MI_back_ and plotted over time (mean intensity as a % background). The mean intensity of the bar histogram was measured at the end of the LY perfusion (4.5 min). Every z-plane was analyzed and plotted separately.

For ring analysis, only the z-plane at 35 µm was analyzed. The ImageJ erosion function was applied 30 times to the whole-spheroid ROI to obtain outer and inner spheroid regions. The mean intensity was measured in these two regions (MI_in_ and MI_out_), normalized (MI(%)) MI_back_ and plotted over time. In addition, the M_in_/MI_out_ ratio was plotted over time. For the bar histogram, the MI(%) and M_in_/MI_out_ ratio were calculated at the end of the LY perfusion (4.5 min).

*Deep-Learning-based Segmentation*: Nuclei images (DAPI for 3D-FT and DRAQ5 for spheroids) were pre-processed by deleting the nuclei of the fibroblast (KRT14 negative region). Nuclei in the keratinocyte region were then segmented using CellPose (V2.2), a deep-learning-based instance segmentation tool. A custom-trained model was developed for each keratinocyte cell line as follows: at least 10 images were pre-segmented with CellPose (using the “nuclei” model) and manually corrected to generate output label masks that were then used in a “human-in-the-loop” approach to generate the custom-trained model. Polygonal ImageJ ROI files defined the overall analysis mask. Cell-pose precomputed nuclei masks were used to identify cell objects. In datasets containing a fibroblast-core mask, nuclei overlapping this region by ≥ 20% were discarded. To capture the peri-nuclear context, a narrow zone surrounding each nucleus was generated using Euclidean distance transform and watershed segmentation, restricted to within two pixels of the nucleus. For each cell, the mean and standard deviation of the fluorescence intensity were calculated across the nuclear region and the cell zone (CZ = nucleus + peri-nuclear zone). The centroid of each nucleus was used to compute distances to structural boundaries, with distances to the spheroid hull reported as positive inside and negative outside. Analogous distances to the fibroblast-core boundary were also determined. These values were used to assign cells to three regions inside the spheroid: Region 1 (surface zone, 0–20 µm from the spheroid hull), Region 2 (inside spheroids, > 20 µm from the hull and not overlapping the fibroblast core), and Region 3 (fibroblast-adjacent, within −20 to 0 µm of the core boundary). The nuclear morphology was characterized by boundary-based estimates of the major and minor axis lengths, aspect ratios, and eccentricity. The perpendicular axis at the centroid was compared with the shortest vector from the centroid to the spheroid surface, and the acute angle (0°−90°) between these vectors was defined as the relative orientation, quantifying nuclear alignment with respect to the spheroid boundary. All analyses were performed in Python using NumPy, Pandas, SciPy, scikit-image, OpenCV, tifffile, and related libraries. Quantitative analysis of the segmentation performance was conducted with the segmentation and detection measures used in the cell tracking challenge (Ulman et al., 2017). As ground truth, for each time point and cell type raw pictures were manually annotated by a biological expert.

### 2.8 Statistics

Statistical significance was calculated using GraphPad Prism 10 software, as described in the figure legends. All data were tested for normal distribution using the Kolmogorov-Smirnov or Shapiro–Wilk normality test. The following statistical tests were applied: two-way ANOVA with multiple comparisons (corrected with Tukey-Report multiplicity adjusted P (0.05 (95% confidence interval)-Output (P value style *0.032, **0.0021, ***0.0002, ****<0.0001)); ordinary one-way ANOVA; T-test unpaired (Non-Parametric Mann Whitney/Kolmogorov, Parametric-Same SD).

## 3 Results

### 3.1 Generation of full thickness skin models and micro-skin spheroids

**FIGURE 1:**
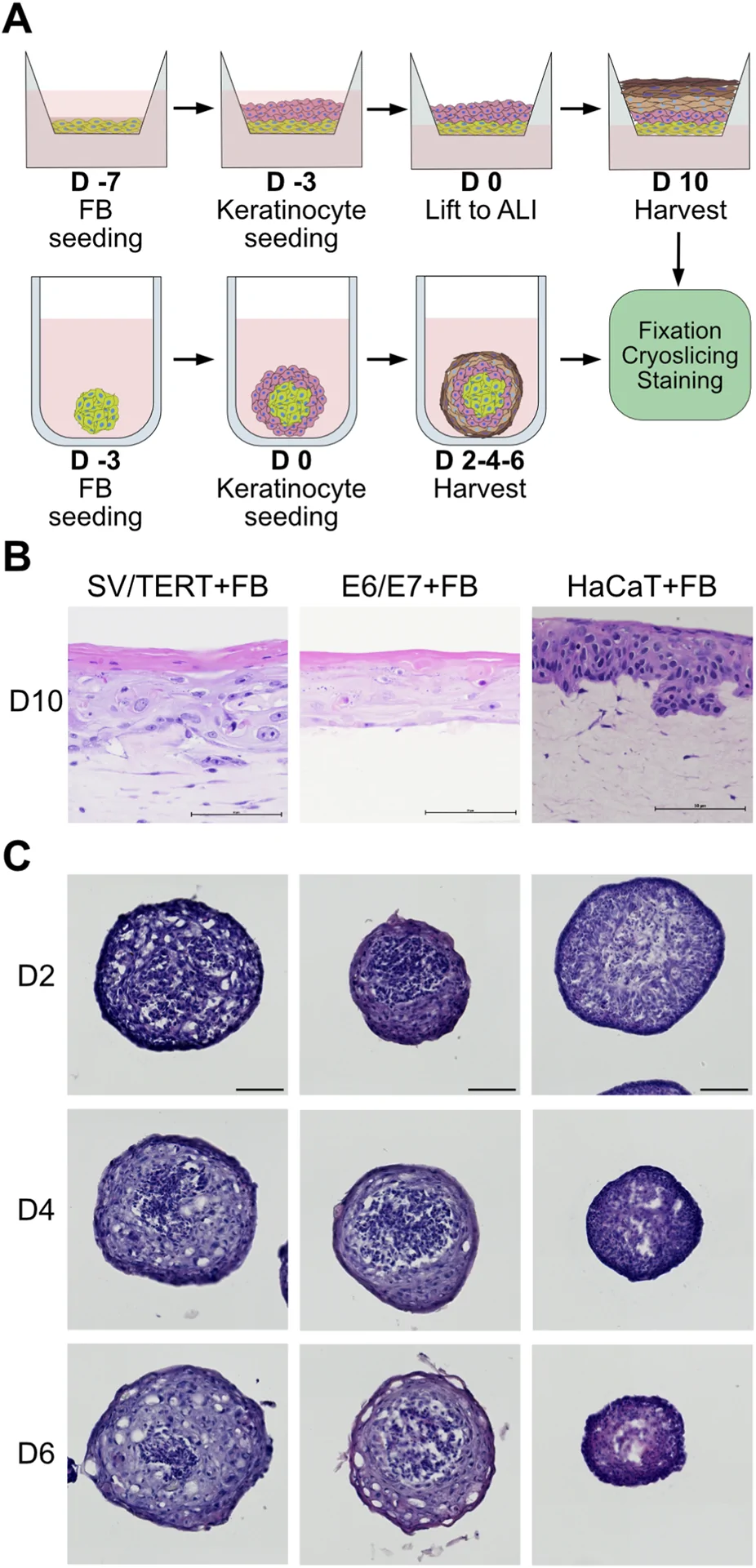
Morphological stratification is observed in both FTSM and spheroid-based dermal-epidermal cell models. **(A)** Schematic illustration of the generation of FTSM (upper panels) and spheroids (lower panels). Initially, fibroblasts were seeded, either on collagen-coated transwell inserts (FTSM) or in ULA plates (spheroids). This was followed by addition of keratinocytes, either 4 days (FTSM) or 3 days (spheroids) later. FTSM were air lifted after 3 additional days and then grown at the air-liquid interface (ALI) for 10 more days before harvesting. Spheroids were cultivated submerged for 2, 4, or 6 days before harvesting. **(B,C)** Representative H&E profiles of FTSM paraffine slices **(B)** and spheroid cryoslices **(C)**. Scale bars for FTSM and spheroids, 50 µm and 100 μm, respectively.

We aimed to develop a spheroid-based dermal-epidermal skin model with different keratinocyte lines and evaluate their physiological significance by comparing them to corresponding ALI-based full-thickness models (FTSM) in terms of differentiation and barrier formation. Spheroid cocultures were generated in ULA plates, whereas FTSM were produced using air-liquid interface culture (ALI) (Idrees et al., 2021). In both systems, human CCD-1137Sk foreskin fibroblasts were first seeded, followed after 3 days (Figure 1A) by addition of keratinocytes from three different cell lines, NHK-SV/TERT, NHK-E6/E7, or HaCaT. While the FTSM were air-lifted, spheroids were cultivated submerged in culture media (Figure 1A). HE staining showed that all FTSM formed an organized epidermis with signs of stratification and a dermal-like fibroblast layer of varying thickness. NHK-SV/TERT and NHK-E6/E7 in FTSM showed a higher degree of differentiation and stratification, with an obvious outermost corneal layer (Figure 1B). Conversely, keratinocytes in HaCaT FTSM were not well organized and showed no layers with a granular or cornified appearance (Figure 1B). In NHK-SV/TERT and NHK-E6/E7 spheroids, a fibroblast core could be clearly distinguished at all time points from the outer keratinocyte layers because of the different nuclear morphologies (Figure 1C). In contrast, fibroblast nuclei in HaCaT spheroids appeared smaller on day 2 and almost disappeared at later stages (Figure 1C). Interestingly, NHK-SV/TERT and NHK-E6/E7 spheroids maintained or slightly increased their size during the observation period, whereas HaCaT spheroids progressively decreased in size (Figure 1C). Altogether, this suggests the occurrence of cell death, primarily of fibroblasts and keratinocytes, in HaCaT spheroids, but not in the other coculture spheroids. In support of this, the area of fibroblasts and keratinocytes decreased over time in HaCaT spheroids, whereas it was constant or even increased in NHK-SV/TERT or NHK-E6/E7 spheroids. All spheroids showed a denser peripheral layer of cells; however, internal stratification was less evident in HaCaT than in NHK-SV/TERT or NHK-E6/E7 spheroids, especially on D4 and D6 (Figure 1C). In particular, NHK-E6/E7 spheroids showed more distinct cell layers, with small, well-defined cells close to the fibroblasts, and progressively flatter cells toward the periphery. By day 6, a densely packed outer layer with a few nuclei was suggestive of stratum corneum (Figure 1C). In summary, in both FTSM and spheroids, NHK-SV/TERT and NHK-E6/E7 keratinocytes reconstituted a multilayered structure, suggesting stratification and likely even cornification, but this was not evident with HaCaT cells.

### 3.2 Cellular differentiation and stratification are pronounced in NHK-FTSM

**FIGURE 2:**
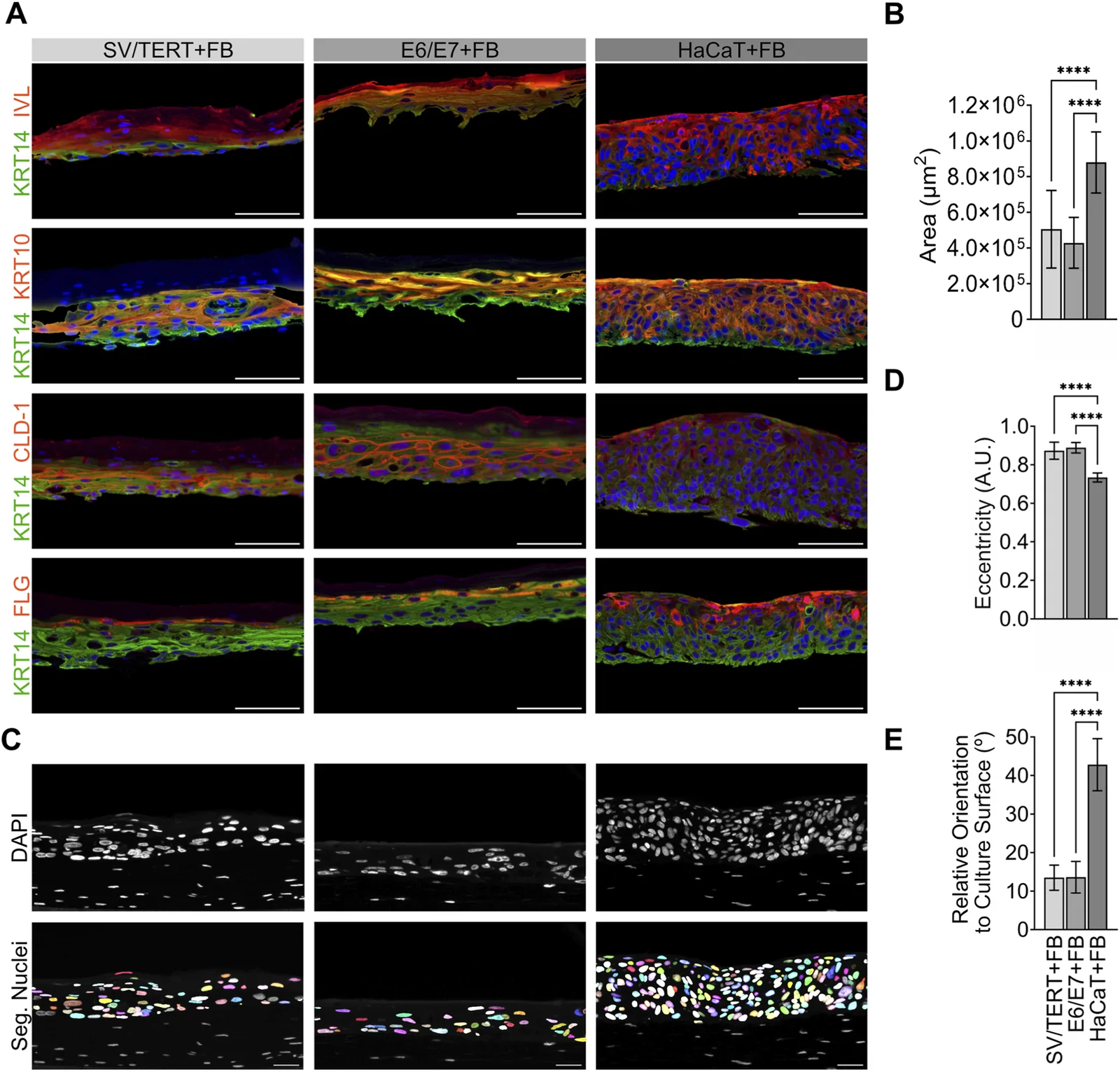
FTSM generated from NHK-E6/E7 or NHK-SV/TERT keratinocytes show stratification and differentiation, which is largely missing in HaCaT models. FTSM composed of fibroblasts and NHK-SV/TERT, NHK-E6/E7, or HaCaT keratinocytes were grown, harvested 10 days post ALI, fixed, sliced and stained for different protein markers, i.e., KRT14, IVL, KRT10, CLD-1, and FLG, as well as DAPI. This was followed by widefield fluorescence microscopy. **(A)** Representative images of FTSM upon immunostaining, DAPI shown in blue, other markers and their color codes as indicated. For a better visualization of the keratinocytes, the fibroblast regions were omitted. Scale bars, 50 µm. **(B)** Plot of the area of the epidermal region. The keratinocyte region was defined based on the KRT14 signal for the lower border, and LY (not shown in this image) for the upper border. **(C)** Representative images of nuclei (upper panels, DAPI) and overlay with the colored segmentation masks of keratinocyte nuclei (lower panels). Scale bars, 20 µm. **(D)** Bar chart of keratinocyte nuclei eccentricity. Mean ± SD (N = 4 experiments). For a value close to 1, the nuclei are elliptic, lower values describe more roundish nuclei. **(E)** Graph depicting the orientation of the nuclei long axis relative to the upper FTSM border. Mean ± SD (N = 16 experiments). A small angle indicates a parallel orientation of the nuclear long axis to the outer border. Two-way ANOVA in **(B)**, one-way ANOVA in **(D,E)**. ****p < 0.001.

To investigate the differentiation and stratification of keratinocytes in FTSM, the expression of involucrin (IVL), cytokeratin 10 (KRT10), claudin 1 (CLD-1), and filaggrin (FLG) was analyzed using immunofluorescence after cryosectioning (Figure 2A). While KRT14 was mainly detected in the basal layer in all models, KRT10 and IVL only showed correct localization in NHK-SV/TERT and NHK-E6/E7 FTSM. In contrast, in the HaCaT model, both markers revealed overlapping expression across the epidermis, and FLG was detected in a broad peripheral band (Figure 2A). In NHK-SV/TERT and NHK-E6/E7 skin equivalents, the tight junction marker claudin-1 (CLD-1) showed good cell membrane staining in the intermediate region, as expected for tight junction formation in the stratum granulosum (Figure 2A). Conversely, in HaCaT FTSM, CLD-1 staining was more diffuse and not confined to the cell borders, indicating no or incomplete barrier formation (Figure 2A). In summary, these data suggest superior epidermis-like differentiation and stratification in the FTSM produced with NHK-SV/TERT and NHK-E6/E7 compared to those made with HaCaT cells. As reduced differentiation often goes along with increased proliferation, it was fitting that HaCaT FTSM were much thicker overall (Figure 2B). In the epidermis, fully differentiated keratinocytes display flattened nuclei, whose main axis is oriented parallel to the border of the stratum corneum (Selby, 1957; Gdula et al., 2013). To study the nuclear morphology of keratinocytes, we developed an automated analysis pipeline based on deep learning segmentation to quantify nuclear shape and orientation. Quantification of nuclei features revealed a significantly reduced eccentricity of HaCaT nuclei compared with the more elongated nuclei of the other cell lines (Figures 2C,D). Furthermore, while the nuclei of NHK-SV/TERT or NHK-E6/E7 keratinocytes were aligned parallel to the culture surface (lower values of relative orientation), such an orientation was absent in the HaCaT nuclei (Figure 2E). In summary, our data confirm previous observations (Jahn et al., 2024), providing strong quantitative evidence of a superior quality of skin models made from immortalized NHK cell lines compared to HaCaT cells in terms of stratification and differentiation.

### 3.3 Lucifer Yellow assay suggests the formation of a functional barrier in NHK-FTSM

**FIGURE 3:**
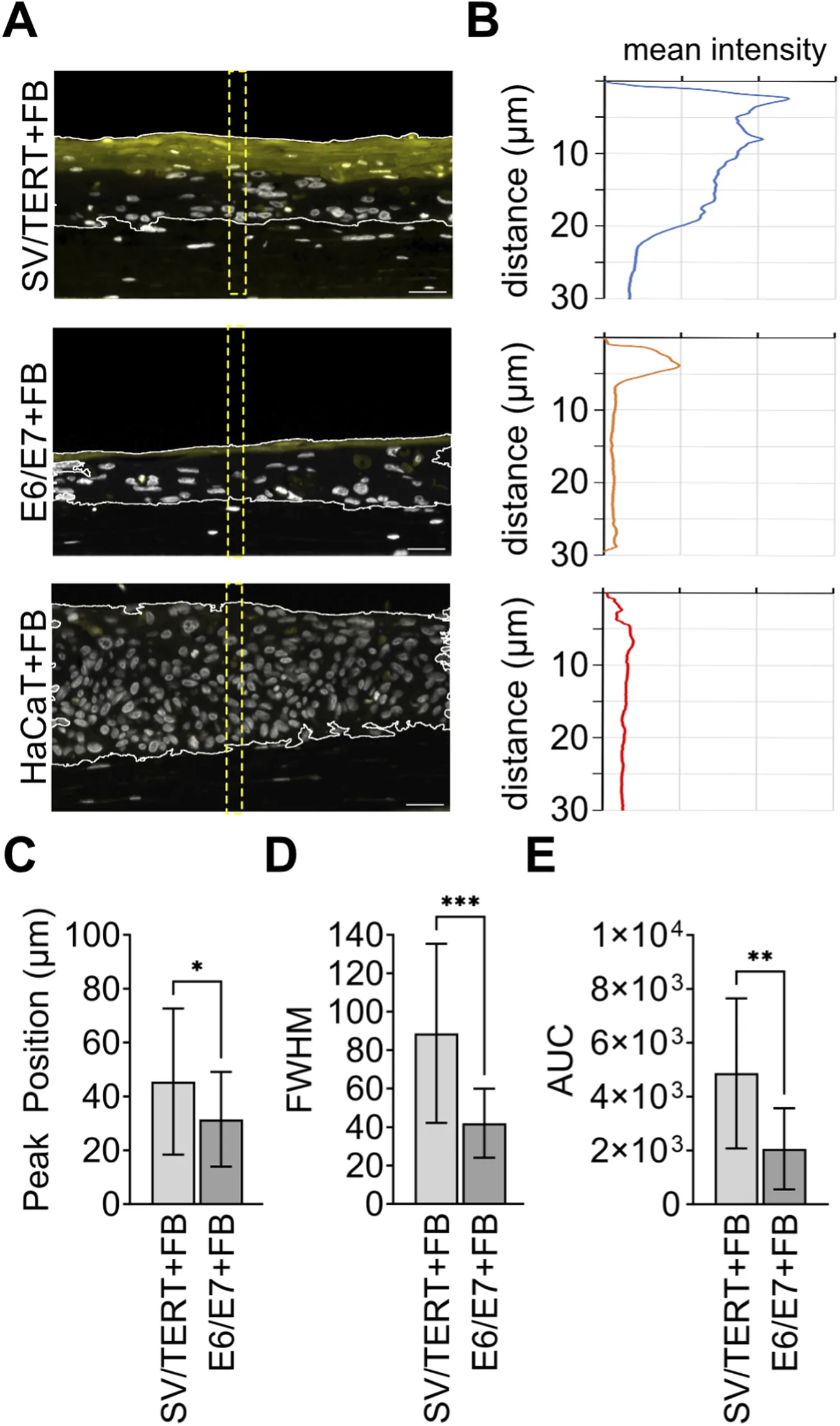
Barrier function is present in FTSM with NHK-E6/E7 and NHK-SV/TERT keratinocytes but not with HaCaT. FTSM composed of fibroblasts and NHK-SV/TERT, NHK-E6/E7, or HaCaT keratinocytes were grown, harvested 10 days post ALI, exposed for 1 h to LY, washed and then fixed, sliced and stained against nuclei (DAPI). **(A)** Representative images of FT models after LY incubation, fixation, and slicing. Nuclei, gray; LY, yellow. Scale bars, 20 µm. **(B)** Plot profiles depicting the mean intensity of the LY signal versus the depth (distance from the upper border), measured in the rectangular yellow dashed regions indicated in **(A)**. **(C–E)** Quantitative analysis of LY staining results. Graphs show mean values ± SD for LY fluorescence peak position (distance from the upper border) **(C)**, full-width half maximum (FWHM) **(D)**, and the area under the curve (AUC) of the plot profile (all, N = 16) **(E)**. Significance was assessed with Mann-Whitney test for peak position and unpaired t-test for FWHM and AUC. The HaCaT FTSM model is not included in the graphs, since these showed no visible LY signal.

Next, using the Lucifer yellow (LY) penetration assay, we tested the functionality of the stratum corneum-like barrier in our FTSM. In the presence of a proper barrier, LY penetrates only slightly between corneocytes but not into the deeper layers. As depicted in Figure 3A, LY was retained in the upper regions of NHK-SV/TERT and NHK-E6/E7 FTSM, but not in HaCaT cells, in agreement with previous observations (Boelsma et al., 1999; van Drongelen et al., 2014; Smits et al., 2017). Usually, LY images are shown as proof of the presence of a functional barrier; however, differences may occur in penetration depth. Therefore, we aimed to quantify LY-staining characteristics by developing an image analysis pipeline. The plot profiles of LY intensity were calculated in the keratinocyte region (Figures 3A,B). This provided quantitative information on the depth and extent of dye penetration, which could be related to the structure and functionality of different keratinocyte layers. Deeper penetration of LY was detected in NHK-SV/TERT than in NHK-E6/E7 FTSM. Plot profile analysis showed, on average, a deeper localization of the peak intensity (Figure 3C), broader penetration window (Figure 3D), and higher LY total intensity (Figure 3E) in NHK-SV/TERT than in NHK-E6/E7 FTSM. HaCaT FTSM did not show any LY signal, as the dye was not retained upon wash-out; therefore, they were not considered for the quantitative analysis (Figure 3B). In summary, these results suggest that the two NHK lines, but not HaCaT FTSM, developed a functional barrier. Furthermore, significant differences in LY diffusion were detected between NHK-SV/TERT and NHK-E6/E7 FTSM, which might reflect the distinct thicknesses and structures of the cornified and granular layers.

### 3.4 NHK-spheroids reproduce important features of epidermal stratification

**FIGURE 4:**
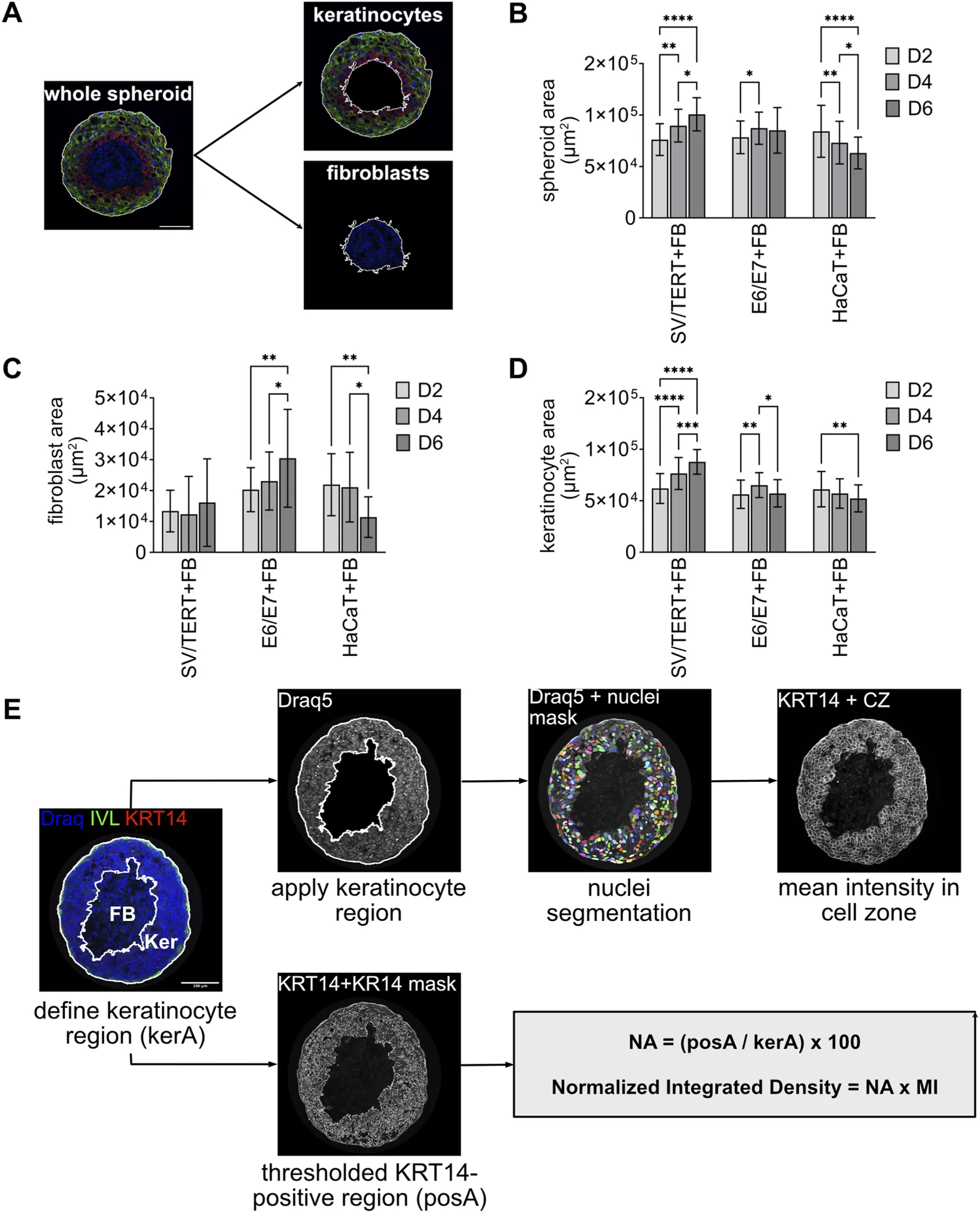
Molecular stratification is well developed in fibroblast-keratinocyte spheroids made with NHK but not with HaCaT keratinocytes. Spheroid coculture models composed of fibroblasts and NHK-SV/TERT, NHK-E6/E7, or HaCaT keratinocytes were grown and then harvested on days 2, 4, and 6 post coculture. Subsequently, spheroids were washed, fixed, sliced, and stained against nuclei (DAPI), KRT14, and IVL. **(A)** Representative image of a spheroid cryoslice immunostained for KRT14 (red) and IVL (green). Nuclei, blue. The white borders indicate the regions selected as indicated. The areas of these regions were calculated with Fiji and the mean values ± SD were plotted of **(B)** total spheroid area, **(C)** fibroblast area, **(D)** keratinocyte area. Statistics used two-way ANOVA (Turkey´s multiple comparison test) comparing for each cell line the different days after spheroid formation (D2, D4, D6) (N = 40). **(E)** Schematic representation of the procedure to obtain segmented keratinocyte nuclei. Images were preprocessed to define and apply the keratinocyte region (to eliminate the fibroblast nuclei). Then, the nuclei were segmented with CellPose and masks were applied to the other channels to measure the mean intensity for each cell. In addition, the KRT14-positive keratinocyte region was used to measure the Normalized Integrated Density.

To gauge whether epidermal stratification occurred in fibroblasts/keratinocyte spheroids, we performed comparable immunostaining experiments on spheroid cryoslices at different time points. Similar to what was observed in Figure 1B, the immunofluorescence images corroborated the presence of a fibroblast core surrounded by a keratinocyte region (Figure 4). Quantitative analysis of the spheroid total areas (see Figure 4 for technical description) revealed that HaCaT spheroids decreased over time, while NHK-SV/TERT spheroids became larger and NHK-E6/E7 spheroids remained stable (Figure 4B). Looking at the individual compartments, the dermal core decreased over time in HaCaT spheroids, remained stable in NHK-SV/TERT spheroids, and increased in NHK-E6/E7 spheroids (Figure 4C). The epidermal portion decreased in HaCaT spheroids, increased in NHK-SV/TERT spheroids, and was essentially unaltered in NHK-E6/E7 (Figure 4D). Overall, only HaCaT spheroids showed an apparent loss of cells in both compartments.

To better understand the contribution of ECM deposition and cell death processes to the shrinkage of HaCaT coculture spheroids, fibroblast-HaCaT spheroids were fixed at days 2, 4, and 6, and stained for collagen IV or cleaved caspase 3, together with nuclei and IVL. As depicted in Supplementary Figure S1, this confirmed the shrinkage of spheroids and the enrichment of IVL in the spheroid rim. While collagen IV was concentrated in the spheroid core on days 2 and 4, it was present throughout at day 6 and its integrated density increased at this time point. Cleaved caspase 3, instead, peaked at day 4 and its signals were present in all spheroid regions. Together, these data suggest that a lack of ECM deposition is not the principal reason for the observed cell death and shrinkage of the spheroids. Furthermore, while the rise of cleaved caspase 3 at day 4 coincides with the major loss of fibroblast cells, it is also compatible with a reduced number of HaCaT cells.

**FIGURE 5:**
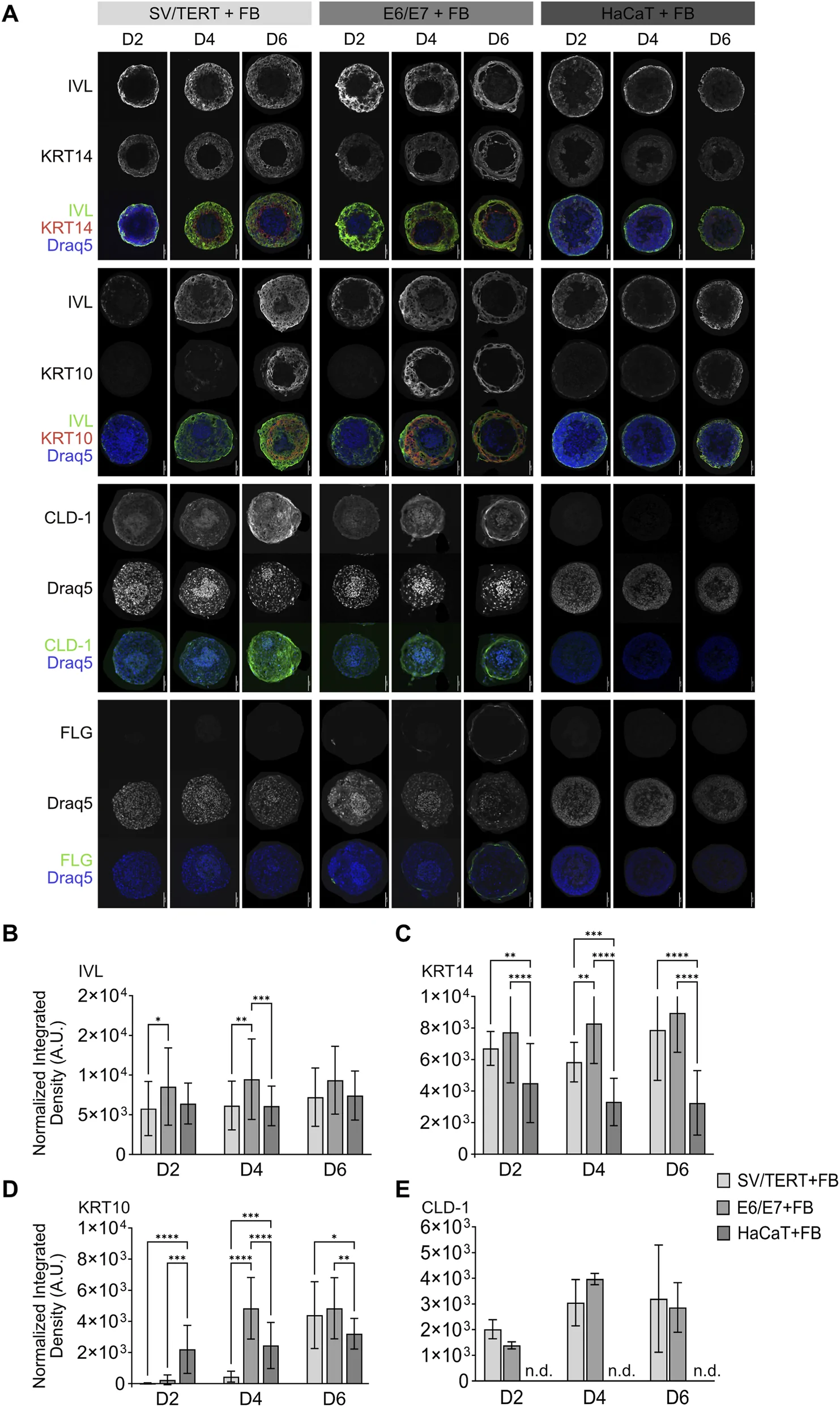
NHK-E6/E7 and NHK-SV/TERT spheroids show well-developed molecular stratification and mature over time, while HaCaT coculture spheroids exhibit weak stratification and deteriorate. Spheroid coculture models composed of fibroblasts and NHK-SV/TERT, NHK-E6/E7, or HaCaT keratinocytes were grown and then harvested on days 2, 4, and 6 of coculture. Subsequently, spheroids were washed, fixed, sliced, and stained against nuclei (Draq5) as well as for markers IVL, KRT14, KRT10, CLD-1, and/or FLG. **(A)** Representative confocal images of cryoslices. Single-channel images of immunostained markers depict fluorescence intensities in gray, as indicated. Merge panels use color codes as indicated. Scale bars, 100 µm. **(B–E)** Graphs of the normalized mean integrated densities ± SD at days 2, 4, and 6 for indicated markers. Statistics used Tukey´s multiple comparison test comparing, for each day, the 3 different cell lines (N between 20 and 30).

To gain a deeper insight into the differentiation features and progression of the three spheroid models, a quantitative analysis was performed for the markers KRT14 and KRT10 (Figure 4A, both red), IVL, FLG, and CLD-1 (Figure 5A, all green) over the three observation time points. Confocal microscopy and data analysis revealed several notable differences between spheroids of different keratinocyte lines, as well as with culture time. While KRT14, KRT10, and IVL were detected in all spheroid types, CLD-1 and FLG showed proper expression and distribution only in NHK-E6/E7 spheroids (Figure 5A). Their expression increased over time and became most evident on day 6, with FLG located in the outer spheroid rim and CLD-1 just beneath (Figure 5A), similar to the observation in FTSM (Figure 2A). In HaCaT spheroids, KRT10 and IVL were present in the entire keratinocyte region, but were enriched in the outermost zone. In contrast, in spheroids made from NHK-E6/E7 or NHK-SV/TERT cells, these markers showed a distinct distribution that was absent in the layer of KRT14-positive cells adjacent to the fibroblast core (Figure 5A). While the IVL extended up to the spheroid border, KRT10 was mainly present in the central region and was absent in the outer spheroid rim (Figure 5A). Quantitative analysis was performed by calculating the normalized mean integrated density, which considers both the mean intensity and area covered by the signal (Figure 4E). Quantification showed that, while IVL did not vary significantly over time and only slightly between the spheroid types (Figure 5B), KRT14 was much less abundant in HaCaT spheroids than in both other spheroid types (Figure 5C). Among all samples, NHK-E6/E7 spheroids showed the highest expression of both IVL and KRT14 at all time points, suggesting that they have well-defined basal and differentiated keratinocyte layers. Interestingly, while KRT10 remained essentially unaltered at all time points in HaCaT spheroids, it markedly increased over time in the other spheroids, being expressed earlier in NHK-E6/E7 (day 4) compared to NHK-SV/TERT (day 6) (Figure 5D). Finally, CLD-1 was measurable only in NHK keratinocyte spheroids, where it showed a trend toward increased expression from days 2–6 (Figure 5E). Overall, these data suggest a wide gap between NHK lines and HaCaT spheroids. Similar to what was observed in FTSM, NHK-E6/E7 spheroids showed the best recapitulation of epidermal differentiation and stratification, as suggested by the expression patterns of KRT10, CLD-1, and FLG.

### 3.5 Single-cell analysis corroborates superior stratification of NHK-spheroids

**FIGURE 6:**
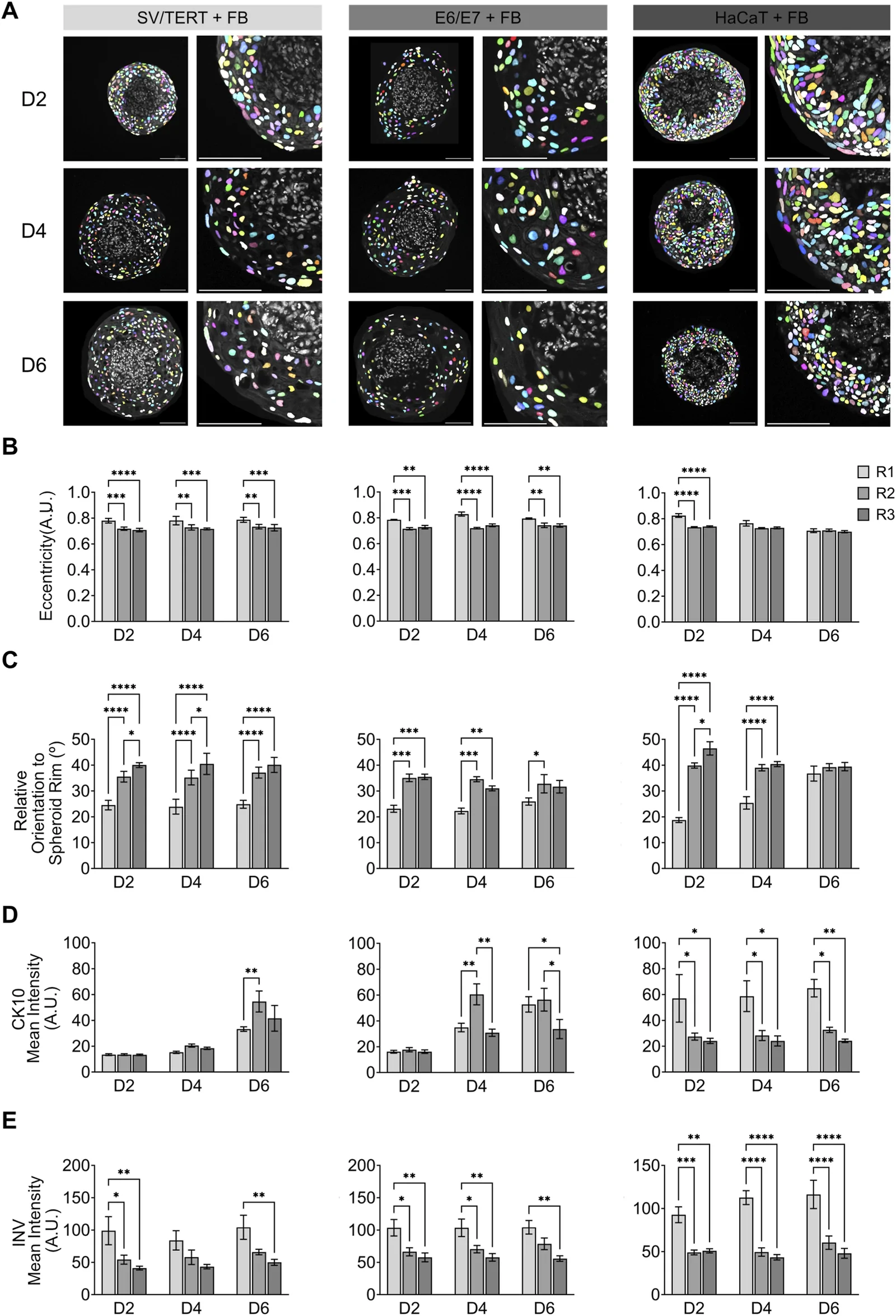
Single-cell analysis of nuclei features and marker localization confirm superior stratification of NHK-E6/E7 and NHK-SV/TERT over HaCaT coculture spheroids. Spheroid coculture models composed of fibroblasts and NHK-SV/TERT, NHK-E6/E7, or HaCaT keratinocytes were grown and then harvested on days 2, 4, and 6 of coculture. Subsequently, spheroids were washed, fixed, sliced, and stained against nuclei (Draq5) as well as for markers, IVL and KRT10. Keratinocyte nuclei or marker intensities were automatically segmented and quantified. **(A)** Segmentation masks of keratinocyte nuclei (colored) were superimposed onto Draq5 raw images (gray). For each cell line, confocal images of spheroids taken at days 2 (D2), 4 (D4), or 6 (D6) were taken. Left panels show representative overview images, right panels their higher magnification. Scale bars, 100 µm. **(B–E)** For each spheroid, the segmented nuclei and cell zones were grouped based on their position. R1: 0–20 µm from the outer spheroid border. R3: 0–20 µm from the fibroblast core. R2: between R1 and R3. Bar plots represent the mean of 4-6 biological replicates ± SD. Each biological replicate comprehends 8–10 spheroids, each spheroid slice has 200–400 cells. Statistics used two-way ANOVA comparing R1, R2, and R3. **(B)** Graphs of nuclear eccentricity: higher values represent more elliptic nuclei. **(C)** Graphs of nuclei relative orientation: lower values represent nuclei parallel to the outer border. **(D,E)** Graphs of the mean intensity in the cell zone for KRT10 **(D)** or IVL **(E)**.

The previous analysis showed that NHK-E6/E7 and NHK-SV/TERT keratinocytes in spheroids have a higher and more stable expression of differentiation markers compared to HaCaT cells; however, regional differences and features of cell morphology were not considered. To better describe the stratification and the ongoing differentiation process, we aimed to gain information at the single-cell level by linking marker expression with cell position and nuclei features. For this purpose, we developed a single-cell analysis pipeline. First, keratinocyte nuclei were automatically segmented using a deep-learning algorithm (CellPose) (Figure 4A). The precision of the segmentation result was tested against a manually segmented ground truth. This revealed a pixel-wise precision ranging from 0.874 to 0.975 (SEG score, minimal score 0, maximal score 1) and an instance-wise detection precision ranging from 0.886 to 0.995 (DET score, minimal score 0, maximal score 1) (Supplementary Figure S2). Next, a program code was developed to retrieve the nuclei orientation versus the outer spheroid border and nuclei eccentricity. Finally, these features were related to the positions of the cells within the spheroid, obtained by measuring the distance of the nuclei to the outer rim and to the keratinocyte/fibroblast interface. The keratinocytes were then segregated into three different regions based on their relative positions: a surface region (R1), a core region (R3), and an intermediate region (R2) (see Material and Methods). Regarding eccentricity, single-cell analysis showed that on day 2 in all spheroid types, the nuclei located in R1 had a significantly higher mean value than the other regions, indicating a more elongated shape (Figures 6A,B). This strongly suggests a location-specific adaptation of keratinocyte morphology, with the outer cells displaying more elongated nuclei, a feature of terminal differentiation similar to that observed in NHK-FTSM (Figure 3D). This difference in eccentricity persisted in NHK-E6/E7 and NHK-SV/TERT spheroids over time but was lost in HaCaT spheroids after day 2, suggesting a loss of differentiated cells at later time points. These findings were corroborated by the analysis of nuclear orientation. In NHK spheroids, at all the time points analyzed, the nuclei in R1 were more aligned to the outer border than those in R2 and R3 (Figure 6C). Conversely, in HaCaT spheroids, R1 nuclei showed a significant difference in alignment only on day 2 (Figure 6C).

Single-cell analysis with regional clustering was used to probe the stratification of differentiation markers. For HaCaT spheroids, both IVL and KRT10 expression were the strongest in R1 cells, and this was constant over time (Figures 6D,E). In contrast, KRT10 was almost negligible on day 2 for the other two spheroid types. Its expression was first observed on day 4 in NHK-E6/E7 and day 6 in NHK-SV/TERT spheroids (Figures 6D,E). Notably, KRT10 was concentrated in the middle region (R2), similar to FTSM. Finally, IVL was mostly enriched in R1 of all spheroids at all time points (Figure 6E), but in contrast to HaCaT cells, it showed a more gradual decrease toward the spheroid core in NHK-E6/E7 and NHK-SV/TERT spheroids (Figure 6E). In summary, these data further support the finding that immortalized keratinocyte spheroids are superior to HaCaT spheroids in terms of epidermis-like organization and stratification.

### 3.6 Live-imaging of Lucifer Yellow diffusion suggests the formation of a functional barrier in NHK-spheroids

**FIGURE 7:**
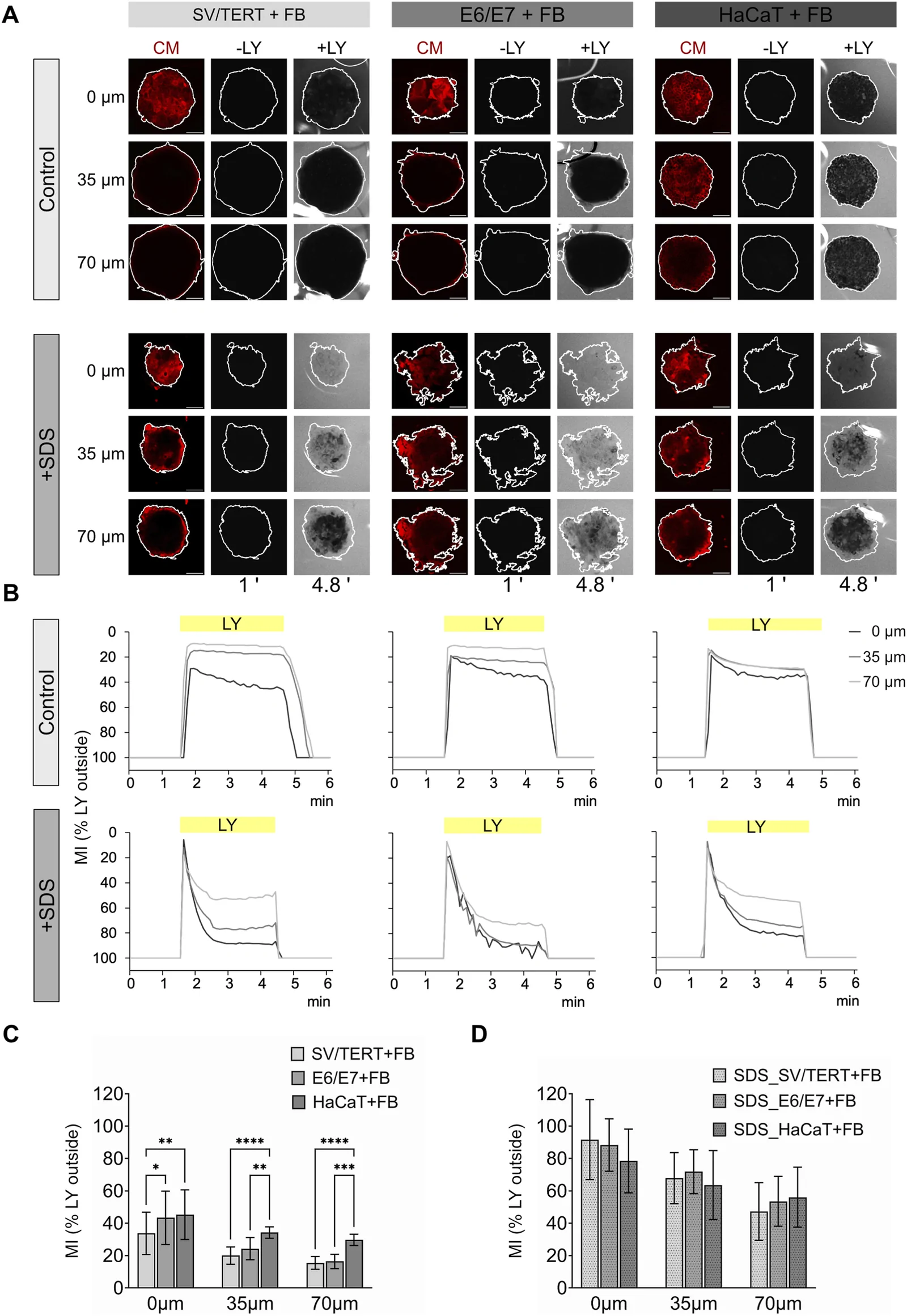
Lucifer Yellow diffuses deeper into coculture spheroids made with HaCaT compared to those with immortalized keratinocytes. **(A)** Representative images in control or upon SDS pretreatment, at different depths from the spheroids’ outer borders, as indicated (0-35-75 µm). Left panels for each cell type: CellMask (CM) fluorescence, used to define the spheroid region (white outline). Central and right panels for each cell type: LY fluorescence in the absence (-LY) and in the presence (+LY) of the dye in the perfusion solution. Images were taken at the times indicated below the bottom panels. White outlines (CM positive) define the spheroid region. Scale bars, 100 µm. **(B)** Time plots of LY fluorescence signal in the spheroid, at different depths, as indicated. The mean intensity was calculated in the spheroid region and normalized to the region outside the spheroid (MI (% LY outside)). In the absence of LY, the spheroid and the background are similarly dark (100%). Upon LY perfusion, the outer region becomes brighter than the spheroid, thus the spheroid fluorescence is only a fraction of the background. **(C,D)** Bar plots represent the mean of MI (% LY outside) ± SD, measured at the end of LY perfusion (4.8 min). Statistics used two-way ANOVA, comparing spheroids of different cell types for distinct depths and in the absence **(C)** or presence of SDS **(D)** (N = 10–22).

To investigate the final differentiation into corneocytes and the formation of a functional barrier in spheroids, similar to what was observed in FTSM, we built upon our previous experience with live spheroid confocal imaging under perfusion (von Molitor et al., 2020) to set up a new protocol that allows time-course measurements of spheroids while applying LY (Figure 7). In this configuration, while LY penetration should be precluded by an existing barrier, it may diffuse inside the spheroid and be retained if the barrier is absent. To define the spheroid region, we used the cell-permeant dye CellMask (CM, Figure 7A). Live-labelled spheroids were imaged at three different z-depths (0, 35, and 70 µm) to evaluate the ability of LY to diffuse into deeper spheroid regions. Spheroids were first perfused with a control solution (-LY), and then with LY (+LY), followed by washout (Figures 7A,B). LY perfusion led to a sudden increase of fluorescence in the solution surrounding the spheroid and, with a small delay, also within the spheroid. The fluorescence measured in the spheroids was normalized to the fluorescence outside and plotted over time (Figure 7B). This showed that the relative change of LY fluorescence was larger at the outer level (0 µm) of NHK-SV/TERT and NHK-E6/E7 spheroids than at the deeper levels, while in HaCaT spheroids all levels showed a similar behavior (Figure 7B). This suggests that LY diffused deeper into HaCaT than into NHK spheroids. At 35 and 70 µm depths, the amount of LY fluorescence was significantly larger in HaCaT spheroids than in those of the other two cell lines (Figure 7C). To corroborate the functional barrier formation, SDS pretreatments are usually employed in FTSM (Smits et al., 2017; Niehues et al., 2018; Capallere, 2021). In our study, SDS pretreatment abolished the differences in LY penetration between the three spheroid types at all depths, strongly suggesting compromised barrier integrity in NHK spheroids under these conditions (compare Figures 7B,D).

**FIGURE 8:**
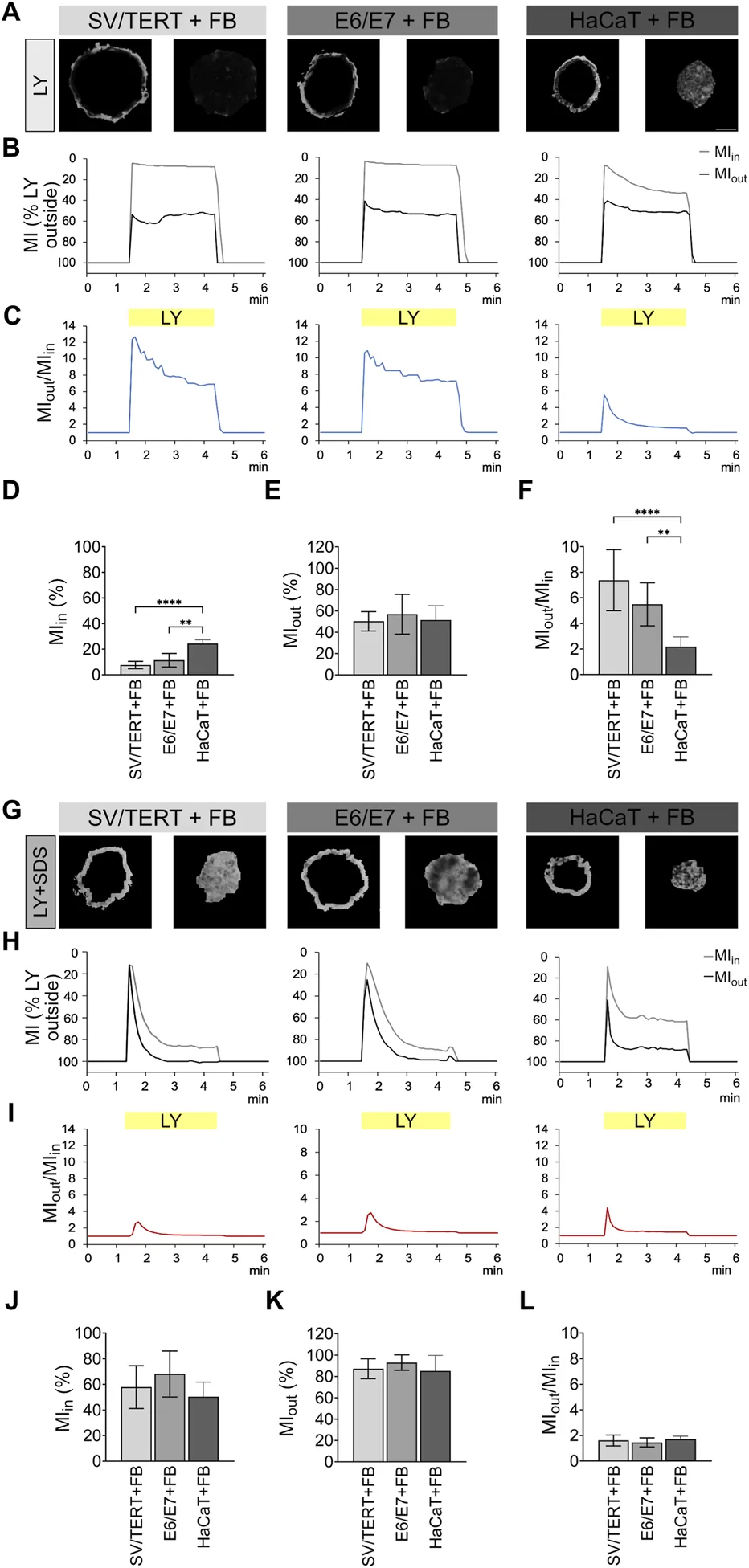
Lucifer Yellow diffuses into the core of HaCaT spheroids, but is hindered in the other spheroids. Spheroid coculture models composed of fibroblasts and NHK-SV/TERT, NHK-E6/E7, or HaCaT keratinocytes were grown for 4–6 days and then live-imaged with confocal time-lapse microscopy. During imaging, spheroids were perfused first in control and then with LY at the time indicated in the plots (yellow bars). Where indicated, they were preincubated with SDS. **(A–F)** Perfusion experiment in the absence of SDS. Coculture type as indicated on top. **(A)** Representative confocal images of coculture spheroids at a depth of 35 μm at 3 min after LY perfusion. Left and right panels of each image pair show only the LY fluorescence intensity in the spheroid border and center, respectively. LY fluorescence outside the spheroids was clipped for better visibility. For full images, see Figure 9. Scale bar, 100 µm. **(B,C)** Graphs show time plots of representative experiments. LY mean intensities in the spheroid regions are depicted as percent of LY fluorescence outside the spheroid (MI (% LY outside)). Time plot of LY mean intensity at the spheroid border (MI_out_, dark gray) and in the center (MI_in_, light gray) **(B)** and of their ratio (MI_out_/MI_in_) **(C)**. **(D–F)** Bar plots represent the means of MI (%) ± SD, as measured in the inner **(D)** and outer regions **(E)** and the mean of their ratio, MI_out_/MI_in_ **(F)**. **(G–L)** LY-perfusion experiment upon SDS pretreatment. Coculture type as indicated on top. **(G)** Representative confocal images of coculture spheroids at a depth of 35 μm at 3 min after LY perfusion. **(H,I)** Graphs of LY mean intensities of spheroid border (MI_out_, dark gray) and center (MI_in_, light gray) as percent of outside LY fluorescence **(H)** and MI_out_/MI_in_ ratio **(I)**. Yellow bar, denoted LY, indicates perfusion period of LY. **(J–L)** Bar plots, mean of MI% ± SD, as measured in the inner **(J)** and outer regions **(K)** and the mean MI_out_/MI_in_ **(L)**. Statistics used two-way ANOVA **(B,C,F,G)** or Kruskal-Wallis nonparametric test with Multiple comparison **(D,H)** (N = 13–17).

As LY diffusion into the spheroids can be measured not only in z-depth but also radially, we quantified the amount of LY fluorescence at the outer border compared to the inner spheroid region at one z-plane. For this purpose, the data were reanalyzed by defining an outer region for each spheroid, i.e., reflecting a potentially cornified zone, and a core region, i.e., the rest of the keratinocytes and fibroblasts (see Materials and Methods). Upon LY perfusion, NHK-SV/TERT and NHK-E6/E7 spheroid core regions remained essentially devoid of LY signals, while LY diffused into the inner parts of HaCaT spheroids (Figure 8A). Quantitative analysis confirmed that LY fluorescence in the spheroid core (MIin) was consistently higher in HaCaT spheroids than in the two NHK spheroid types (Figures 8B–D), whereas fluorescence in the outer region (MIout) was comparable between all spheroid types (Figure 8E). In summary, this led to a significantly higher MIout/ MIin ratio in NHK-E6/E7 and NHK-SV/TERT spheroids, compared to HaCaT spheroids (Figure 8F). Notably, SDS pretreatment led to a massive diffusion of LY into the cores of all spheroid types and thus ablated any difference previously observed (Figures 8G–L). In summary, LY live-imaging experiments showed that this hydrophilic dye was much more excluded from the cores of NHK-SV/TERT and NHK-E6/E7 spheroids, suggesting the existence of a functional barrier in these spheroids, similar to what observed in FTSM. This is also in agreement with previous observations in fibroblast/keratinocyte spheroids obtained with either HaCaT or primary keratinocytes, where, upon long LY incubation, only HaCaT spheroids were massively loaded (Xie et al., 2025). Our LY diffusion experiments complemented the marker expression analysis to corroborate the assumption that the relevant structural, cellular, and biochemical components, which contributed to the generation of the functional barrier, were only present in NHK spheroids. Furthermore, our new experimental setting for LY diffusion appeared to be suitable not only for testing the presence of a barrier, but also for providing additional spatial information and temporal kinetics to enable a more detailed study of the barrier properties and small differences therein.

## 4 Discussion

This study yielded strong evidence that coculture spheroids with fibroblasts and NHK-SV/TERT or NHK-E6/E7 keratinocytes were capable of fully differentiating into a mature epidermal structure, similar to FTSM obtained from the same cells. Thus, we propose these spheroids as promising alternative 3D skin models. Furthermore, we developed and applied new quantitative analysis tools that allowed for a detailed comparison of keratinocyte maturation, stratification, and barrier function across both FTSM and spheroid formats.

For the immunostained cryoslices, we developed an analysis pipeline that allows the quantification of marker expression and nuclear features with single-cell precision, and that combines this information with the position of the cells within the spheroids. This strongly suggested that NHK-SV/TERT and NHK-E6/E7 coculture spheroids display progressive maturation and stratification, in line with what we observed in comparable FTSM. In contrast, HaCaT spheroids shrank over time, underwent disorganization, and did not fully mature. A further sign of full epidermal maturation of NHK-SV/TERT and NHK-E6/E7 coculture spheroids was the evidence for the presence of a functional barrier, as tested by the LY diffusion assay. Interestingly, NHK-based spheroids blocked LY penetration, while with HaCaT cells the dye diffused into the spheroid core. These data are in line with the FTSM results and with previous epidermal ALI-based skin models generated with the same cell lines. In summary, our data support the generation of a stratified epidermis and a functional barrier in FTSM and spheroids composed of fibroblasts plus either NHK-SV/TERT or NHK-E6/E7, but not HaCaT cells.

Thus, owing to their matched morphological and functional features with FTSM, NHK coculture spheroids introduced here arguably represent a miniaturized 3D skin model with distinct advantages over classical human skin equivalents. Their application in dermatological research may increase when the following benefits are considered: First, production can be scaled, considering the much lower number of cells required for spheroid generation compared to organotypic skin models. Second, they can be handled in multi-well plates, thereby enabling automated processing and reducing culture volumes. Third, given their rapid formation and differentiation, dermal-epidermal spheroids reduce the time required to generate corresponding skin equivalent cultures by at least half, significantly saving time. Fourth, since spheroids are processed throughout in submerged culture, drug treatments should be easier to perform and require smaller amounts than in ALI cultures.

Next, regarding the use of HaCaT cells to generate 3D skin models, this study provides a detailed temporal and spatial analysis of differentiation, stratification, and functional barrier, which indicates the similarity between spheroids and ALI-based skin models with HaCaT cells and extends what has already been reported in the literature. Indeed, it was previously shown that organotypic skin cultures with HaCaT keratinocytes initially form a multilayered epithelium that increasingly disorganizes and undergoes senescence (Ryle et al., 1989; Boelsma et al., 1999). In particular, while KRT10 expression was restricted to the uppermost layer after 1 week of culture in the skin equivalent, it was later ubiquitously expressed in all cell layers (Boelsma et al., 1999). This was in contrast to primary keratinocytes, which showed that KRT10 expression was restricted to the suprabasal layers in FTSM (Idrees et al., 2021). Similarly, our results in HaCaT-based FTSM show ubiquitous expression of KRT10 in all layers and the presence of lobulated and irregular nuclei also in the outermost layer, while in native skin the keratinocytes of the stratum corneum are flattened and anucleated (Boelsma et al., 1999). HaCaT cells easily form spheroids either alone or with a fibroblast core (Klicks et al., 2017; Klicks et al., 2019; Schäfer et al., 2021; Strzyga-Łach et al., 2024; Vitacolonna et al., 2024a). Fibroblast/HaCaT spheroids reproduced some epidermal features, including KRT10 and KRT14 expression; however, KRT10 localization was incorrect (Klicks et al., 2019). Additionally, complex stratification has not been reported and barrier function has not been tested (Klicks et al., 2019; Vitacolonna et al., 2024a). Our marker expression analysis in HaCaT coculture spheroids suggested an advanced differentiation state, but missing correct stratification and the formation of a cornified layer. While on day two, HaCaT nuclei shape and orientation suggested a kind of organization of the cell layers, HaCaT spheroids underwent increasing disorganization and cell death, making this model unsuitable for long-term cultivation experiments. Additionally, single-cell analysis clearly displayed incorrect localization of KRT10, while the lack of FLG and CLD-1, and of a functional barrier, revealed the absence of a stratum corneum. Thus, our data clearly define the limitations of HaCaT based dermal/epidermal spheroids.

Conversely, some immortalized NHK cells have already been shown to have higher physiological relevance than HaCaT cells. Along these lines, another immortalized NHK line was previously reported to generate FTSM displaying a higher similarity to primary keratinocytes, with correct localization of KRT10 in the stratum granulosum and spinosum (Van Drongelen et al., 2014). In our case, FTSM generated with NHK-SV/TERT or NHK-E6/E7 displayed all four epidermal layers, according to marker expression. In particular, FTSM with NHK-E6/E7 yielded the best stratification and a marker distribution comparable to that obtained with primary keratinocytes (Jahn et al., 2024). Interestingly, nuclear features, elongation, and parallel orientation also favored adequate layering and terminal differentiation in the two NHK-based FTSM.

Similar features were also observed in NHK coculture spheroids. While IVL expression was more pronounced in the outer region and constant over time, the expression of the other differentiation markers increased at later time points, suggesting progressive differentiation of the keratinocytes during the 6 days of cultivation. Single-cell analysis revealed that KRT10 expression was largely restricted to the central region, which could correspond to the suprabasal layer. This localization was observed in both NHK-based spheroid types, but expression was delayed in NHK-SV/TERT cells. This confirmed a previous speculation that the switch from proliferation to differentiation may take longer in NHK-SV/TERT than in NHK-E6/E7 (Jahn et al., 2024). A more stratified and differentiated phenotype in the NHK-E6/E7 spheroids was also supported by the expression of CLD-1, which was better localized in a thin outer cell layer. Similarly, FLG was exclusively present in NHK-E6/E7-spheroids, where it was restricted to the outermost region, i.e., corresponding to the granular layer. This expression pattern was paralleled by progressive flattening of the keratinocyte region and elongation and alignment of the nuclei with the outer spheroid rim. Moreover, the presence of a functional barrier in NHK-spheroids was suggested by the exclusion of LY in live imaging experiments. Since refractive index mismatches in thicker three-dimensional cell cultures can potentially lead to light attenuation or scattering (Nürnberg et al., 2020), one could argue that this may be one of the reasons for the reduced LY values at 70 µm depth, affecting our interpretation that the data depicted in Figures 7, 8 would reflect different levels of barrier function. However, three main points argue against such a false interpretation. First, comparing LY fluorescence intensities at the same depth between the distinct spheroid types revealed clear differences; i.e., NHK spheroids showed consistently lower LY fluorescence than HaCaT spheroids (Figure 7C). Assuming a similar scattering induced by all three keratinocyte types, this may be explained with a reduced diffusion of LY into NHK spheroids. Second, LY fluorescence intensity rose strongly upon SDS treatment, ablating the differences between spheroid types at all depths (Figure 7D). Third, LY signals were uniform throughout the entire optical plane in HaCaT spheroids (Figure 7A control HaCaT; Figure 8A HaCaT), thus speaking against a major effect of tissue height on signal intensity, at least at the depths tested here. Indeed, if light scattering would obstruct light passage as a function of depth, the LY signal in HaCaT spheroids should become dimmer towards the spheroid center in a given optical plane, due to the spherical nature of the cultures, but this was not the case. Of course, a margin of uncertainty remains, but we believe that these arguments strengthen the hypothesis that the data presented in Figures 7, 8 primarily reflect biological differences in the barrier function of spheroids and not merely an optical artifact. Next, although the experimental constraints did not allow to determine transepithelial electric resistance (TEER) as an additional important readout of a functional barrier, the distribution of CLD-1 and FLG, and the LY exclusion corroborated the occurrence of terminal differentiation, a functional barrier, and a competent stratum corneum, especially in the NHK-E6/E7 spheroids. Given that the usual paradigm for forming a functional barrier involves exposure of the keratinocytes to airlift, we were initially surprised to observe robust exclusion of LY and the proper localization of tight junction and stratum corneum markers. However, evidence for functional barriers was previously also obtained in submerged cultures using primary keratinocytes (Chen et al., 2025) and in skin organoids (Ramovs et al., 2022), suggesting that relevant stratum corneum features can be also obtained in the absence of airlift. Previous work showed the stimulation of ceramide formation and induction of differentiation markers in submerged adherent cultures of primary keratinocytes by acidification, high levels of Ca^2+^, and addition of linoleic acid (Breiden et al., 2007). Although this was not addressed experimentally, our culture conditions, combining a medium with a special and undisclosed mix of growth factors and lipids, the presence of fibroblasts producing extracellular matrix and the U-shaped microwells might have created a local milieu favoring the enrichment of such components, thus triggering cornification reactions in the spheroids. To our knowledge, this is the first evidence that a functional skin barrier can be achieved in a micro-skin spheroid model generated with keratinocyte cell lines, which highlights its similarity to primary keratinocyte spheroids (Xie et al., 2025).

Only a few groups have pursued the generation of spheroids from keratinocytes, other than HaCaT cells. Notably, the presence of a fibroblast core was key to achieving multilayered spheroids (Butt et al., 1996; Xie et al., 2025). Primary keratinocytes, when cultivated in BME droplets, develop an inside-out epidermis model with corneocytes in the core (Agarwal et al., 2023). Spheroids can be generated with primary human neonatal keratinocytes, but with low efficiency, and neither differentiation nor stratification has been proven (Woappi et al., 2020; Woappi et al., 2021). In coculture spheroids generated with iPSC-derived fibroblasts, keratinocytes, and endothelial progenitors, keratinocytes organized around the fibroblast core expressed KRT14 and KRT10, but stratification and cornification were not present (Ebner-Peking et al., 2021). More successfully, micro-skin organoids generated with a fibroblast/collagen core and keratinocytes derived from epiblast cells fully differentiated and developed a functional barrier (Xie et al., 2025). In our case, NHK cells could not form spheroids without fibroblasts but only loose cell aggregates. Instead, stable spheroids were formed in the presence of fibroblast-conditioned medium; however, in the absence of a fibroblast core, the keratinocytes did not show stratification (data not shown). Thus, our dermal-epidermal spheroid cultures with NHK-lines showed a higher level of cell differentiation and layering, adding physiological significance to spheroids used as skin models. In particular, the superior quality of these spheroids was substantiated by the coexistence of basal and differentiating keratinocytes in the same spheroid but in different regions, with KRT14-positive cells mainly present around the fibroblast core, corresponding to the basal layer, and the other markers localized according to a physiological skin differentiation program.

There are several potential use cases and further practical implications for fibroblast/NHK spheroids and for the assays presented in this study. First, considering the applications of confocal live-imaging LY assays, our experimental setting and kinetic analysis allowed for detailed quantification of LY penetration and its time course. Our live-imaging study of dye diffusion into spheroids might open new ways to study the permeation and absorption of drugs, which can be relevant to the development of formulations for transdermal drug delivery (Van Gele et al., 2011; Ribeiro et al., 2025). Several concentrations can be tested through perfusion in a dynamic manner. It also has the potential for high-throughput applications using an automated confocal system. However, ULA plates are not suitable for confocal microscopy because of their thick plastic and concave shapes; thus, they should be substituted with imaging-compatible polymer arrays (Keller et al., 2020; Fois et al., 2024). Perfusion systems should also be automated to allow for defined and reproducible treatments. In a previous work, HaCaT-based dermal-epidermal spheroids were generated with hydrogels in a microfluid assay and used for screening the efficacy of vitamins and peptides and the toxicity of chemical agents. They were also tested for diffusion of CellTracker-Red-CMTPX, which labelled HaCaT cells but did not reach the fibroblast core (Chen et al., 2025). This paper presents a basis for high-throughput screening using skin spheroids and live imaging. Thus, a combination of our NHK-based spheroids with an automated confocal live-imaging and microfluidic system might allow the generation of a high-throughput platform to study the skin barrier integrity and diffusion of fluorescently labelled molecules. NHK-based spheroids might complement pharmacologic, toxicologic, and cosmetic tests that take place in human skin equivalents or in animals *in vivo*, opening the possibility of larger and faster screenings (Mittler et al., 2017; Vitacolonna et al., 2024b).

Second, supposing an improved physiological significance of our NHK-based spheroids, they could be useful for disease modeling. Previously, it was shown that incorporation of melanoma cells into fibroblast/HaCaT spheroids significantly reduced KRT10 enrichment at the spheroid rim. Cytostatic treatment efficiently killed melanoma cells and recovered the peripheral localization of KRT10 (Klicks et al., 2019; Schäfer et al., 2021). It would be interesting to model melanoma in triculture spheroids with immortalized NHK cells to investigate how cancer cells affect differentiation and prove drug efficiency in recovering stratification and barrier function (Barghian Zarnaghi et al., 2025).

Of note, the conclusions in this study were not merely based on qualitative analysis, but were achieved by detailed quantitative information. The advanced segmentation-based image analysis of immunostained cryoslices yielded quantitative single-cell information, i.e., describing the position, nuclear morphology and orientation, and marker expression for each cell. This novel analytical approach, when applied to both spheroids and FTSM, can provide more precise spatial information. In particular, nuclei features can be used as a reliable readout of differentiation and stratification in the case of the skin. Furthermore, this approach can also be used to study differentiation and cell patterning in other tissues and organoids (Gdula et al., 2013; Hughes et al., 2013; Roby and Rauzi, 2025).

Currently, there is a major bottleneck in terms of throughput in our experimental design. Indeed, immunofluorescence microscopy of both skin equivalents and spheroids relies on the tedious and time-consuming cryosectioning process. Thus, to unleash the full performance of spheroid-based experimental studies, it is important to circumvent the need for cryosectioning and instead enable whole-mount immunostaining and 3D analysis, which can be multiplexed and automated. Previously, general protocols for whole-mount immunostaining and optical tissue clearing of 3D cell cultures were devised to meet this need (Nürnberg et al., 2020; Schäfer et al., 2022). However, while the available methods were found to be applicable to HaCaT spheroids (Nürnberg et al., 2020; Vitacolonna et al., 2024a), they were ineffective for NHK-based spheroids (data not shown). This was most likely due to the enhanced barrier function of these spheroids, which did not permit the penetration of antibodies, staining reagents, or light. New clearing protocols for skin spheroid models should be developed based on the results already obtained on skin biopsies (Abadie et al., 2018; Fernandez and Marull-Tufeu, 2019; Tan et al., 2020). Future work should address this challenge in more detail to further improve the benefits and speed at which fibroblast/NHK-based spheroids can address open topics in experimental dermatology.

## 5 Conclusion

In summary, we developed spheroids consisting of fibroblasts and immortalized keratinocytes that mature to robust, stratified, barrier-competent dermal/epidermal structures. Together with our deep-learning–based image analysis tools, these models offer intermediate-complexity, physiologically relevant, and moderately high-throughput platforms for dermatological testing.

## Data Availability

The raw data supporting the conclusions of this article will be made available by the authors, without undue reservation.
