# Supplementary material for: Combining advanced 3D spheroid-based skin models with deep-learning-based image analysis enables in-depth investigation of keratinocyte differentiation and barrier function
Source: Front Bioeng Biotechnol. 2026 Sep 9;14:1887652. doi: 10.3389/fbioe.2026.1887652 (PMC13597799; doi:10.3389/fbioe.2026.1887652)
Supplement: Supplementary file 1 [file Supplementaryfile1.docx]

Supplementary data to manuscript

Combining advanced 3D spheroid-based skin models with deep-learning-based image analysis enables in-depth investigation of keratinocyte differentiation and barrier function

by Tiziana Cesetti et al.

**Figure S1: Fibroblast-HaCaT cocultures exhibit ECM availability and apoptosis in large parts of the spheroids.** Spheroid coculture models composed of fibroblasts and HaCaT keratinocytes were grown and then harvested on days 2, 4, and 6 post coculture. Subsequently, spheroids were washed, fixed, sliced, and stained against nuclei (DAPI), Coll-IV or Cleaved Caspase 3, and IVL. Micrographs show representative IVL and Coll-IV or Cleaved Caspase 3 signals alone (indicated) or in an overlay with nuclei. In overlays, see annotation on top for color code. Scale bars, 100 µm. Bar graphs depict average integrated densities of collagen-IV and cleaved caspase-3 signals as a function of coculture time (N= 10).

**Figure S2: Automated nuclei segmentation shows high fidelity compared to a manual process.** Spheroid coculture models composed of fibroblasts and NHK-SV/TERT, NHK-E6/E7, or HaCaT keratinocytes were grown and then harvested on days 2, 4, and 6 of coculture. Subsequently, spheroids were washed, fixed, sliced, and stained against nuclei (Draq5). After deletion of the fibroblasts core, keratinocyte nuclei were segmented and quantified by either manual segmentation (ground truth, GT) or through an automated algorithm (CellPose), see Materials and Methods. The micrographs depict exemplary raw nuclei signals (uppermost panels) of day 2 spheroids as indicated. The panels below show the corresponding segmented nuclei as obtained by the manual (GT, green) or automated process (CellPose, red). A perfect match is printed in yellow. Scale bars, 100 µm. The table reports pixel-wise (SEG score) and instance-wise (DET score) overlap between GT and CellPose for the different tested sample sets. Both scores can range from zero (no overlap) to 1 (maximal overlap).
